# Fancy Citrus, Feel Good: Positive Judgment of Citrus Odor, but Not the Odor Itself, Is Associated with Elevated Mood during Experienced Helplessness

**DOI:** 10.3389/fpsyg.2016.00074

**Published:** 2016-02-02

**Authors:** Matthias Hoenen, Katharina Müller, Bettina M. Pause, Katrin T. Lübke

**Affiliations:** ^1^Institute of Experimental Psychology, Heinrich Heine University DüsseldorfDüsseldorf, Germany; ^2^Federal Employment Agency GermanyNürnberg, Germany

**Keywords:** citrus, limonene, vanillin, aromatherapy, mood, helplessness

## Abstract

Aromatherapy claims that citrus essential oils exert mood lifting effects. Controlled studies, however, have yielded inconsistent results. Notably, studies so far did not control for odor pleasantness, although pleasantness is a critical determinant of emotional responses to odors. This study investigates mood lifting effects of d-(+)-limonene, the most prominent substance in citrus essential oils, with respect to odor quality judgments. Negative mood was induced within 78 participants using a helplessness paradigm (unsolvable social discrimination task). During this task, participants were continuously (mean duration: 19.5 min) exposed to d-(+)-limonene (*n* = 25), vanillin (*n* = 26), or diethyl phthalate (*n* = 27). Participants described their mood (Self-Assessment-Manikin, basic emotion ratings) and judged the odors’ quality (intensity, pleasantness, unpleasantness, familiarity) prior to and following the helplessness induction. The participants were in a less positive mood after the helplessness induction (*p* < 0.001), irrespective of the odor condition. Still, the more pleasant the participants judged the odors, the less effective the helplessness induction was in reducing happiness (*p* = 0.019). The results show no odor specific mood lifting effect of d-(+)-limonene, but indicate a positive effect of odor pleasantness on mood. The study highlights the necessity to evaluate odor judgments in aromatherapy research.

## Introduction

The strong association of odors with emotions, both on the neurophysiological and on the experience level (e.g., Adolph and Pause, 2012), suggests that odors are effective mood regulators. Indeed, the application of aromatic compounds in order to relieve stress and pain or elevate mood is a common procedure in alternative medicine. Citrus essential oils in particular have been claimed to exert mood enhancing effects (Pimenta et al., 2012). However, studies regarding mood lifting effects of citrus odors show mixed results. In rodents the inhalation of citrus essential oils alleviates stress, and exerts anxiolytic effects (Komiya et al., 2006; Leite et al., 2008; Lima et al., 2013). Likewise, in human’s anxiolytic effects of citrus fragrances have been suggested: Patients waiting for a scheduled appointment at a dental office report reduced anxiety when orange odor is introduced as ambient fragrance (Lehrner et al., 2000, 2005). However, the anxiety reducing effect proved not to be odor specific (Lehrner et al., 2005). Others found that dentist patients’ anxiety level was unaffected by any ambient odor (orange odor vs. apple; Toet et al., 2010). It has further been claimed that treatment with citrus ambient odors normalizes neuroendocrine and immune function in depressive individuals (Komori et al., 1995). Indeed, depressive individuals seem to display a specific preference for citrus fragrances (citral; Pause et al., 2001). Notably, none of the studies reporting mood enhancing effects of citrus odors examined subjective judgments of the odors’ quality, although this has been identified to be a key factor determining the emotional response to odors (Herz, 2009).

Learned helplessness, a negative emotional state which is characterized by a loss of control and negative expectations regarding the future, can be used as a model for depression (Miller and Seligman, 1975). Furthermore, the state effects of helplessness resemble deviations in central odor processing of depressed individuals (Laudien et al., 2006). Learned helplessness can be induced in controlled settings using ecologically valid success–failure manipulations (Nummenmaa and Niemi, 2004; Laudien et al., 2006).

The current study investigates the mood effects of d(+)-limonene (limonene), one of the most prominent compounds in citrus essential oils (characterized as a fresh citrus orange note) within a highly controlled setting. A learned helplessness procedure was used to induce a slightly negative mood, and odor judgments as well as mood ratings were obtained prior to and following the helplessness induction.

Vanillin and diethyl phthalate served as control conditions. The introduction of a vanillin control allowed for disentangling specific odor effects from pleasantness effects, as both limonene and vanillin are generally regarded as pleasant. The diethyl phthalate control served for the discrimination of odor effects from non-specific chemosensory context effects, as diethyl phthalate was the solvent for both limonene and vanillin.

The judgment of an odor’s quality as pleasant or unpleasant essentially affects the emotional response to this odor (Herz, 2009). Furthermore, beliefs about an odor (e.g., regarding an odor as unhealthy) are more important in determining the individual response to that odor than its actual biochemical properties (De Araujo et al., 2005; Laudien et al., 2008). Therefore it is expected that the experienced odor quality and not the odor itself modulates mood.

## Materials and Methods

### Participants

A total of 97 volunteers participated in the experiment. All participants reported to be healthy, and free of neurological or psychiatric conditions. In order to heighten the subjective importance of the cover story (task used for employee selection in the social domain; see Cover Story), only participants working in the social domain (e.g., social worker) or studying a subject related to social sciences (e.g., psychology, educational science) were recruited (see Cover Story). Due to technical problems (*n* = 9) and disbelief in the cover story (*n* = 10) 19 participants were excluded. Of the final sample (*n* = 78), 27 participants (23 females) were included in the diethyl phthalate condition, 26 participants (22 females) were assigned to the vanillin condition, and 25 participants (21 females) were assigned to the limonene condition. Age (*M* = 24 years, *SD* = 7, range 18–59) did not differ between conditions (*p* > 0.90).

The study was conducted in accordance with the Declaration of Helsinki and was approved by the ethics committee of the Faculty of Mathematics and Natural Sciences of the Heinrich-Heine-University Düsseldorf. Participants gave their written informed consent and were compensated with course credit or €15. At the end of the experiment participants were debriefed and informed about the true nature of the study.

### Cover Story

Participants were asked to take part in a study investigating the effects of right brain hemisphere activation on odor habituation. They were informed that they would be working on a computer-based emotional intelligence test, which leads to activation of the right brain hemisphere, while inhaling an odor. It was stated that the emotional intelligence test would usually be applied to test professional aptitude in the social domain (e.g., physiotherapy, social work, or psychotherapy). Participants were told that it was crucial to do their best at the task in order to determine whether they possessed a skill that is important for their profession. The cover story was adapted from Laudien et al. (2006).

### Materials

#### Odors

D-(+)-limonene (97%, Sigma–Aldrich Co.; diluted 1:2 [v/v] in diethyl phthalate [99%, Merck KGaA]), Vanillin (99% Sigma–Aldrich Co.; diluted 1:10 [v/v] in diethyl phthalate [99%, Merck KGaA]), and diethyl phthalate (99%, Merck KGaA) were used as odorants. Odor concentrations of d-(+)-limonene and vanillin were chosen to be perceived as medium intense, and roughly matched for intensity (as judged by working group members).

Odors (3 ml) were dropped on cotton pads, which were placed in gas-washing bottles (100 ml volume). An air operated double diaphragm pump (Tetratec APS 50, Tetra GmbH; volumetric flow rate 14 ml/s) was used to pump ambient air through the gas-washing bottles into an oxygen mask. Air flow was controlled using computer controlled solenoid valves. Separate teflon-tubes (6 mm diameter) were used for each odor. Odors were presented continuously from the beginning of the helplessness induction until the second rating of odor quality (duration: *M* = 19.5 min, *SD* = 2.5 min).

#### Stimuli for the Helplessness Induction Procedure

In order to affect the participants’ emotional state, an unsolvable emotional intelligence test was introduced in the cover story of the experiment (Laudien et al., 2006). A total of 175 faces (Karolinska Directed Emotional Faces System; Lundqvist et al., 1998) were presented in a facial expression assessment task. Of these, 92% were of neutral valence (45.8% neutral, 45.8% surprise), and 8% were of negative valence (fear: 5.7%, sadness: 0.4%, anger: 1.5%, disgust: 0.8%). Stimuli were presented on a 19″ TFT monitor (Terra LCD 4319, Wortmann AG) positioned at 1 m distance using Presentation 14 (Neurobehavioural Systems Inc.).

#### Questionnaires

The effects of the helplessness induction were assessed on the dimensions emotional valence (-4 = negative valence, 4 = positive valence), arousal (0 = low arousal, 9 = high arousal), and dominance (0 = low dominance, 9 = high dominance) using the language-free computerized Self-Assessment Manikin (SAM; Bradley and Lang, 1994). Furthermore, participants indicated their emotional state regarding five basic emotions (anger, disgust, fear, happiness, and sadness) via computerized visual analog scales (length: 18.5 cm, range: 0–100). Odor quality was rated regarding intensity, pleasantness, unpleasantness, and familiarity using pictographic computerized nine level likert-scales, similar to the SAM.

### Procedure

Participants were assigned to the three treatment groups (diethyl phthalate, limonene, vanillin) and were tested separately. At the beginning of each session, the participants indicated their baseline mood, using the SAM and emotion ratings. The participants were then asked to discriminate one deviant odor (the treatment odor) from two distractors (three alternative forced choice), which were presented in a random sequence via the oxygen mask (stimulus duration = 7 s; interstimulus interval = 7 s). Vanillin and limonene were tested against diethyl phthalate, whereas diethyl phthalate was tested against ambient air. The task was repeated five times.

Then, immediately prior to the experiment, participants rated the quality of the treatment odor regarding intensity, pleasantness, unpleasantness and familiarity. During the odor quality ratings the odor was presented continuously in order to match the odor presentation during the actual experiment.

**FIGURE 1:**
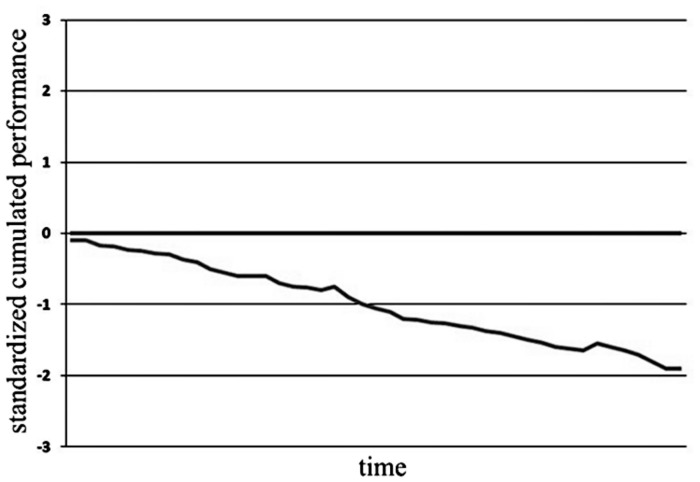
**False performance feedback.** The decreasing line was introduced as the actual performance of the participant over time, the horizontal line (0) was introduced as average performance. The axes’ labels were not presented to the participants.

Helplessness was induced using a facial expression classification task. A total of 264 pictures of faces were presented briefly but supraliminally (100 ms duration). Pictures were presented in random order, but no picture was repeated directly after it was shown for the first time. Participants were asked to evaluate whether these faces express either a negative or positive emotion. This was an unsolvable task due to the mostly neutral facial expressions of the stimuli presented. Decisions had to be made by mouse click within a 3-s interval. Participants were advised not to skip any pictures because all unrated faces would be counted as false. In order to induce helplessness, participants received false feedback regarding their performance over time after every 6th decision (duration: 4 s; number of feedbacks: 44, see **Figure 1**). Starting from the beginning feedback indicated “below average” and progressively worsening performance, reaching a score indicating a “quite poor performance” after the 21st trial. The feedback graphs and the meaning of the scoring were explained to the participants before testing. This procedure was followed by the participants rating their mood and judging the odor’s quality a second time.

Throughout the entire session, the mean ambient temperature was kept at 24°C (*SD* = 1 °C). A complete session lasted between 48 and 77 min.

### Statistical Analysis

The effects of the helplessness induction procedure and the odor exposition on perceived odor quality (intensity, pleasantness, unpleasantness, familiarity) were analyzed using a 3 × 2 split-plot ANOVA with the factors odor (diethyl phthalate, vanillin, limonene) and time (prior to helplessness induction [T1], after helplessness induction [T2]). Bonferroni-corrected *t*-tests were used as *post hoc* tests (α = 0.050/3 = 0.017).

Mood ratings (SAM ratings: emotional valence, arousal, dominance; basic emotion ratings: anger, disgust, fear, happiness, and sadness) were subjected to the same ANOVA. In order to correct for multiple tests, the significance level for the ANOVAs was bonferroni corrected to α = 0.050/8 = 0.006.

Effects of odor hedonics on mood were assessed using a linear multivariate regression including both mean odor pleasantness and unpleasantness as predictors for difference values of emotional valence, dominance, anger, and happiness (T2 – T1). Emotional valence, dominance, anger, and happiness were chosen because these ratings proved to be affected by the helplessness induction procedure, as evident from the ANOVAs (main effect of time, see Results). Predictors were entered in the model simultaneously. In order to correct for multiple tests, the significance level for the regression models was bonferroni corrected to α = 0.050/4 = 0.013.

## Results

### Odor Perception

The treatment groups did not differ in their ability to detect the target odor [χ^2^(2) = 2.13, *p* = 0.347]. The target odor was correctly detected at least four times (chance level < 0.05) by 69% of the participants.

Limonene (*M* = 5.5, *SD* = 1.8) was perceived as more intense than diethyl phthalate [*M* = 3.4, *SD* = 1.7; *t*(50) = 4.22, *p* < 0.001] and vanillin [*M* = 4.3, *SD* = 1.1; *t*(49) = 2.88, *p* = 0.006]. The intensity of vanillin and diethyl phthalate did not differ significantly after bonferroni-correction [*t*(51) = 2.15, *p* = 0.036; main effect odor: *F*(2, 75) = 11.18, *p* < 0.001]. During the course of the experiment strong habituation effects were evident: All odors were perceived as more intense before the helplessness induction (*M* = 5.0, *SD* = 2.4) than after the helplessness induction [*M* = 3.7, *SD* = 1.9; main effect time: *F*(2, 75) = 26.12, *p* < 0.001]. In detail, diethyl phthalate [*t*(26) = 2.59, *p* = 0.016] and limonene [*t*(24) = 4.76, *p* < 0.001] were rated as less intense after the helplessness induction, whereas ratings for vanillin did not differ between measurements [*t*(25) = 1.59, *p* = 0.124].

Odors did not differ regarding pleasantness [*F*(2, 75) = 0.03, *p* = 0.972] or unpleasantness [*F*(2, 75) = 3.05, *p* = 0.053]. All odors were rated as more unpleasant (*M* = 2.2, *SD* = 1.4) after compared to before the helplessness induction [*M* = 2.7, *SD* = 2.2; main effect time: *F*(1, 75) = 5.27, *p* = 0.024].

Vanillin (*M* = 5.3, *SD* = 2.2) and limonene (*M* = 5.6, *SD* = 1.9) were rated as more familiar than diethyl phthalate [*M* = 3.3, *SD* = 2.1; vanillin vs. diethyl phthalate: *t*(51) = 3.20, *p* = 0.002; limonene vs. diethyl phthalate: *t*(50) = 4.10, *p* < 0.001; main effect odor: *F*(2, 75) = 9.02, *p* < 0.001]. Diethyl phthalate was rated as even less familiar after the helplessness induction (*M* = 2.7, *SD* = 2.3) than before the helplessness induction [*M* = 4.0, *SD* = 2.5; *t*(26) = 2.7, *p* = 0.010], while the familiarity of limonene and vanillin did not vary over time [interaction odor × time: *F*(2, 75) = 3.52, *p* = 0.035]. For an overview of the odor quality ratings see **Table 1.**

**Table 1:** Descriptive values of odor quality ratings.

	Diethyl phthalate				Vanillin				Limonene			
			
	T_1_		T_2_		T_1_		T_2_		T_1_		T_2_	
						
	*M*	*SD*	*M*	*SD*	*M*	*SD*	*M*	*SD*	*M*	*SD*	*M*	*SD*
Intensity	4.0	2.4	2.8	1.7	4.6	1.7	3.9	1.6	6.5	2.2	4.4	2.1
Pleasantness	4.7	2.7	4.6	2.4	5.0	2.1	4.5	2.1	5.1	2.1	4.4	2.4
Unpleasantness	1.9	1.2	2.6	1.7	1.9	1.5	2.4	2.1	3.0	1.8	3.3	2.5
Familiarity	4.0	2.5	2.7	2.3	5.2	2.9	5.3	2.2	5.6	1.9	5.6	2.1


*T_1_, before helplessness induction, T_2_, after helplessness induction, *M*, mean, *SD*, standard deviation.*

### Mood Ratings

The helplessness induction was successful. Regardless of odor condition, participants indicated they were in a more negative mood (emotional valence), more submissive (dominance), angrier (anger) and less happy (happiness) after compared to before the helplessness induction (all *p*s < 0.001; see **Table 2** for ANOVA results; see **Tables 3** and **4** for descriptive statistics). Odors had no effect on mood (all *p*s ≥ 0.067; see **Table 2**).

**Table 2:** Effects of odor and helplessness induction (time) on mood.

	Odor			Time			Odor × Time		
			
	*F*(2, 75)	*P*	$\eta_{p}^{2}$	*F*(1, 75)	*p*	$\eta_{p}^{2}$	*F*(2, 75)	*p*	$\eta_{p}^{2}$
Valence	0.77	0.466	0.020	55.94	<0.001	0.427	0.01	0.987	<0.001
Arousal	0.79	0.458	0.021	7.34	0.008	0.089	1.06	0.508	0.018
Dominance	0.86	0.426	0.022	18.31	<0.001	0.196	0.42	0.662	0.011
Anger	0.04	0.953	0.002	49.50	<0.001	0.398	0.08	0.921	0.001
Fear	0.06	0.934	0.002	0.58	0.450	0.088	2.64	0.078	0.066
Disgust	2.17	0.121	0.055	0.40	0.533	0.005	0.77	0.467	0.020
Happiness	0.67	0.515	0.018	77.96	<0.001	0.510	0.25	0.779	0.007
Sadness	0.36	0.940	0.010	4.06	0.047	0.051	2.81	0.067	0.070


*Bonferroni adjusted significance level: α = 0.006.*

**Table 3:** Descriptive values of mood ratings (SAM).

	Diethyl phthalate				Vanillin				Limonene			
			
	T_1_		T_2_		T_1_		T_2_		T_1_		T_2_	
						
	*M*	*SD*	*M*	*SD*	*M*	*SD*	*M*	*SD*	*M*	*SD*	*M*	*SD*
Valence	1.9	1.3	0.4	2.1	1.5	1.3	0.0	1.4	1.9	1.4	0.4	1.7
Arousal	4.5	1.9	5.1	2.1	4.2	1.3	4.4	1.5	4.2	1.2	5.0	1.6
Dominance	5.6	1.7	5.1	1.6	5.7	1.5	5.2	1.5	6.2	1.3	5.5	1.3


*T_1_, before helplessness induction, T_2_, after helplessness induction, *M*, mean, *SD*, standard deviation.*

**Table 4:** Descriptive values of mood ratings (basic emotions).

	Diethyl phthalate				Vanillin				Limonene			
			
	T_1_		T_2_		T_1_		T_2_		T_1_		T_2_	
						
	*M*	*SD*	*M*	*SD*	*M*	*SD*	*M*	*SD*	*M*	*SD*	*M*	*SD*
Anger	7.6	13.5	28.4	25.1	7.7	10.9	25.7	26.8	8.0	13.3	27.6	25.2
Fear	7.1	12.5	10.3	16.6	11.2	22.0	7.2	15.8	7.8	10.6	12.4	16.1
Disgust	7.1	11.1	5.8	10.2	7.1	13.1	7.8	14.9	11.2	14.9	15.2	22.2
Happiness	49.3	19.5	35.8	23.4	53.9	22.7	39.0	18.7	54.6	14.8	42.4	18.8
Sadness	6.3	11.5	13.2	17.6	14.2	23.9	11.7	20.0	5.9	11.4	13.0	21.2


*T_1_, before helplessness induction, T_2_, after helplessness induction, *M*, mean, *SD*, standard deviation.*

A model using odor pleasantness and odor unpleasantness as predictors^1^ explained 12.3% (*R*^2^) of the variance in the change of happiness over the course of the helplessness induction [*F*(2, 75) = 5.28, *p* = 0.007]. Participants reported a smaller reduction of happiness the more pleasant [β = -0.268, *t*(75) = 2.48, *p* = 0.019] and, by trend, the less unpleasant they rated the odor [β = 0.191, *t*(75) = 1.74, *p* = 0.087]. A similar effect was found for emotional valence: Participants reported a more negative valence after the helplessness induction the more unpleasant the odor was rated [β = 0.250, *t*(75) = 2.23, *p* = 0.028]. However, after bonferroni-correction the overall model predicting emotional valence is not considered significant [*F*(2, 75) = 3.60, *p* = 0.032].

The odors’ pleasantness and unpleasantness cannot predict the change in dominance or anger ratings over the course of the helplessness induction, after bonferroni-correction is applied (see **Table 5**).

**Table 5:** Parameters for regression model with odor pleasantness and unpleasantness as predictors.

	Overall model			Odor pleasantness			Odor unpleasantness		
			
	*R*^2^	*F*(2, 75)	*p*	β	*t*(75)	*p*	β	*t*(75)	*p*
Valence	0.088	3.60	0.032	-0.121	1.08	0.285	0.250	2.23	0.028
Dominance	0.010	0.37	0.691	-0.050	0.43	0.667	0.077	0.66	0.511
Anger	0.043	1.67	0.196	0.022	0.194	0.846	-0.201	1.75	0.084
Happiness	0.123	5.28	0.007	-0.264	2.40	0.019	0.191	1.74	0.087


*Bonferroni adjusted significance level for the overall model: α = 0.013.*

ANCOVAs including the factors of the original ANOVAs (odor and time) and odor pleasantness as well as odor unpleasantness as covariates support the previous ANOVAs’ results: Mood ratings still are unaffected by odor (all *ps* > 0.4, except for sadness ratings, odor × time: *p* = 0.074).

Also the results of the regression analysis are replicated: Participants show a smaller happiness reduction the more pleasant [time × pleasantness: *F*(1, 73) = 5.42, *p* = 0.023] and the less unpleasant they rated the odor [time × unpleasantness: *F*(1, 73) = 3.97, *p* = 0.050]. Further, participants reported a more negative valence after the helplessness induction the more unpleasant the odor was rated [time × pleasantness: *F*(1, 73) = 5.05, *p* = 0.028].

## Discussion

The current study aimed at investigating whether the odor of limonene would be especially potent in preventing the induction of negative mood by a learned helplessness procedure. However, the present results indicate that limonene, like the control odors (vanillin, diethyl phthalate), was ineffective at preventing negative mood, even though the current design achieved a statistical power of 0.97 (medium effect sizes assumed [*f* = 0.25, Cohen, 1988]). Moreover, the observed null effect is independent of the application of a bonferroni-correction. Thus, the current results are in line with Toet et al. (2010), who also could not show a mood lifting effect of orange odor, and seem to contradict those studies showing positive effects of orange odor on mood (Lehrner et al., 2000, 2005).

On the other hand, the effectiveness of the helplessness induction varied between individuals in accordance with their ratings of the odors’ pleasantness. In detail, the more pleasant the odors were rated, the less successful (in terms of a smaller decrease in happiness) the helplessness induction was. Moreover, it is possible to assume that these differences in perceived odor pleasantness actually caused the mood stabilizing effect (instead of happiness affecting odor pleasantness): Odor pleasantness was rated the same prior and after the helplessness induction. Therefore, the respective pleasantness judgment can be considered as having been evident before any changes in mood occurred.

Taken together, this pattern indicates that mood lifting effects of limonene and vanillin can primarily be attributed to their pleasantness and not to their specific aromatic profile or chemical structure. These results are in line with studies showing effects of pleasant odors on the autonomic nervous system congruent with positive mood (e.g., Alaoui-Ismaïli et al., 1997; Heuberger, 2001). Thus, odors might indeed work as mood enhancers, as long as they are perceived as pleasant. As learned helplessness, which was utilized within the current study to induce negative mood, is regarded as an etiologic model for depression, the current work especially underlines the close connectivity between odors and emotions in the context of depression (Pause et al., 2003; Schablitzky and Pause, 2014). Our results further suggest that being exposed to pleasant odors might attenuate the experience of negative mood in a situation typically involved in the development of depressive symptomatology. Pleasant odors might therefore be an additional support in the treatment of depressive symptoms.

It could be speculated that specific mood enhancing effects of limonene might have been prevented by its potentially irritating properties (Larsen et al., 2000). However, a reduction in perceived intensity over the course of the experiment suggests that the participants showed perceptual habituation. Habituation indicates that the olfactory properties of limonene dominated, as trigeminal stimulation should rather have led to sensitization (Hummel and Kobal, 1999; Hummel, 2000).

It could be argued that the generalizability of the current results might be somewhat limited due to an overrepresentation of females within the sample. However, according to previous studies, gender does not modulate the effects of pleasant and unpleasant odors on mood (Marchand and Arsenault, 2002), rendering a similar gender bias within the current results unlikely. Further, as women were equally distributed among the odor groups, possible odor effects could not have been confounded by gender.

So far, research examining the potential of odors – and citrus odors in particular – to prevent negative mood has yielded inconclusive results. The current data suggest that such conflicting results might be related to odor pleasantness judgments varying between individuals and from study to study, rendering the respective odors either effective or ineffective mood enhancers. Therefore, the current study is in line with studies showing that judgments about an odor are more important in determining the response to it than its biochemical properties (De Araujo et al., 2005; Laudien et al., 2008) and Herz’s (2009) conclusion, that the effects of aromatherapy in humans may primarily be attributed to psychological effects.

## Conclusion

The current study indicates that odor pleasantness and not limonene itself has a mood enhancing effect. Odor effects in humans are provoked by the individual perception of a particular odor, and not by the intrinsic properties of the odor. Thus, the study highlights the necessity to evaluate the odor judgments of the participants in aromatherapy research.

## Author Contributions

All authors listed, have made substantial, direct and intellectual contribution to the work, and approved it for publication.

## Conflict of Interest Statement

The authors declare that the research was conducted in the absence of any commercial or financial relationships that could be construed as a potential conflict of interest.

## Footnotes

1 Note that ratings for pleasantness and unpleasantness can both be used as predictors in the multivariate regression, since they are not correlated (*r* = -0.174, *p* = 0.129).
